# Plasma Cell Infiltration on Histopathological Samples of Chronic Bone and Joint Infections due to *Cutibacterium acnes*: A series of 21 Cases

**DOI:** 10.7150/jbji.46187

**Published:** 2020-06-23

**Authors:** Alexis Trecourt, Marie Brevet, Anne Champagnac, Anne Conrad, Jérôme Josse, Céline Dupieux-Chabert, Florent Valour, Tristan Ferry

**Affiliations:** 1 Hospices Civils de Lyon, Institut de pathologie multisites des Hospices Civils de Lyon, Site Est et plateforme de pathologie moléculaire, Bron, France.; 2 Université Claude Bernard Lyon 1, Lyon, France.; 3 Hospices Civils de Lyon, Hôpital de la Croix-Rousse, Service de Maladies Infectieuses et Tropicales, Lyon, France.; 4 Centre de Référence des Infections Ostéo- Articulaires Complexes de Lyon, Lyon, France.; 5 Centre International de Recherche en Infectiologie, CIRI, Inserm U1111, CNRS UMR5308, ENS de Lyon, UCBL1, Lyon, France.; 6 Hospices Civils de Lyon, Hôpital Croix-Rousse, Institut des Agents Infectieux, Laboratoire de Bactériologie, Lyon, France.

**Keywords:** *Cutibacterium acnes*, Bone and joint infection, Plasma cells, Plasma cells infiltration, CRIOAc Lyon's criterion

## Abstract

**Introduction:** Histopathological definition of bone and joint infection (BJI) is based on Mirra's criterion (≥ 5 polymorphonuclears (PMNs) per field in 5 high power fields (HPFs)). However, this definition does not seem appropriate for chronic BJIs caused by slow-growing germs such as *Cutibacterium acnes* (*C. acnes*). The aim of this study was to confirm that Mirra's criterion is not adequate for diagnosis of BJIs due to *C. acnes*. The second objective was to determine if plasma cell infiltration could be useful for the diagnosis of chronic BJIs due to *C. acnes*.

**Methods:** We retrospectively selected 25 consecutive patients from 2009 to 2013 with chronic BJIs due to *C. acnes*. Histological analysis was performed on the 21 cases with at least two *C. acnes* positive cultures. In addition of Mirra's criterion, the number of plasma cells (≥5 plasma cells/5 HPFs, defined as “CRIOAc Lyon's criterion”) was implemented in the histopathological analysis. Patients were defined as infected, if at least one of the two criteria were present.

**Results:** According to Mirra's and CRIOAc Lyon's criteria, positive histopathology was observed in 12 (57.1%) and 15 (71.4%) cases respectively. Considering the 9 cases with negative Mirra's criterion, high plasma cell infiltration (≥5 plasma cells per field/5 HPFs) was observed in 5 cases (55.6%), and low plasma cells infiltration (2-5 plasma cells per field/5 HPFs) was observed in 4 other cases (44.4%).

**Conclusions:** Adding CRIOAc Lyon's criterion to Mirra's criterion might restore some histopathological diagnosis of chronic BJIs due to *C. acnes* when a chronic BJI is clinically suspected.

## Introduction

Chronic bone and joint infections (BJIs) are one of the most difficult-to-treat infectious diseases 1, 2, increasing the risk of morbidity and mortality 1. In chronic implant-associated BJI, such as chronic prosthetic-joint infection (PJI), as in fracture-related chronic infection (FRI), such as infected nonunion or chronic osteomyelitis, various bacteria could be involved with different mechanisms of bacterial persistence 1.

The diagnosis of chronic BJI is more complex than in acute setting. According to the International Consensus Meeting (ICM) 2018, the diagnosis of PJI is based on: i) major criteria: two positive cultures of intraoperative specimens with the same organism or presence of a sinus tract; or ii) combination of minor criteria including serum and intraarticular inflammatory parameters, a single positive culture, and positive histopathology 3, 4. The recommended threshold of polymorphonuclears (PMNs) on section of formalin-fixed paraffin embedded (FFPE) tissue for PJI diagnosis, defined as Mirra's criterion, is ≥ 5 PMNs per field in each of 5 high power magnification fields (HPFs; initially described by Mirra et al. at 500x magnification, then described by the Musculoskeletal Infection Society at 400x magnification) 5, 6. The clinical, microbiological, and histopathological diagnostic criteria for FRI are nearly the same as those used for the diagnosis of PJI and have recently been clarified 7, 8. However, some authors suggested that the histopathological threshold is too high for the diagnosis of all arthroplasty infections 9. Indeed, certain microorganisms, especially coagulase-negative staphylococci and *Cutibacterium acnes* (*C. acnes*), could cause a prosthetic infection with a PMN infiltration rate <5 PMNs per field 6. Additionally, Mirra's criterion has low sensitivity to predict the presence of microorganisms in samples from suspected aseptic prosthetic loosening 10.

*C. acnes* is a gram-positive, non-spore-forming, aerotolerant anaerobic, slow-growing bacillus. This bacterium is usually found in the cutaneous, upper respiratory and digestive mucosae flora 11, 12. However, *C. acnes* is also well recognized as an invasive orthopedic pathogen, sometimes difficult to detect 1. Because of its slow growth, this microorganism is mostly responsible for chronic BJIs with few clinical symptoms 1, and needs longer culture times, up to 2 weeks, leading to difficult-to-diagnose infections 12. Moreover, a positive culture with *C. acnes* may be a contamination and can be difficult to interpret 12. The diagnosis of *C. acnes* BJIs could be missed 11, and is frequently delayed, leading to an important risk of implant loosening/instability, chronic pain and treatment failure 12, 13.

The aim of this study was to confirm that Mirra's criterion based on the detection of ≥5 PMNs per field/5 HPFs is not adequate in all chronic *C. acnes* BJIs. Moreover, as acute bone inflammation could shift over time to chronic infiltration of lymphocytes and plasma cells 14, the second objective was to determine if plasma cell inflammation could be a useful criterion for histopathological diagnosis of chronic BJIs due to *C. acnes*.

## Methods

From the prospective ongoing cohort *Lyon BJI cohort study* (NCT02817711) of our regional reference centre called CRIOAc Lyon (http://www.crioac-lyon.fr), we selected consecutive patients with *C. acnes* BJI diagnosed from 2009 to 2013 for whom additional clinical data were collected to determine the management of such infections 15. Initial diagnoses were made based on clinical, microbiological, radiological and pathological data. We retrospectively applied the ICM 2018 criteria for definition of PJI 3, 4 and consensus proposed by Metsemarkers et al. and then adopted by the FRI consensus group for definition of FRIs 8, 16, included 25 patients for whom pathology sample slides at the time of surgery for *C. acnes* BJI were kept and still available. Final histopathological analysis was performed only on patients with PJI or FRI defined by the presence of at least two *C. acnes* positive monobacterial cultures on separate deep samples collected intraoperatively from the site of infection, in order to prove that an infection to *C. acnes* was occurring 3, 4, 8, 16. Each patient had between 2 and 8 intraoperative samples for culture, the number of samples to be taken was left to the surgeon's discretion.

Histopathological analysis was performed on FFPE 3µm Hematoxylin-Eosin-Safran slides, reviewed collectively by three pathologists. Inflammation criteria were described in hot spots; they included Mirra's criterion (≥ 5 PMNs per field/5 HPFs) and some additional criteria: low PMN infiltration (0, <2, 2-5 PMNs per field/5 HPFs), low plasma cell infiltration (0, <2, 2-5 plasma cells per field/5 HPFs), granulation tissue, necrosis, giant cells and the presence of fibrin. Based on Mirra's criterion to define a BJI and because the histopathological threshold must be easily achievable by pathologists, we have implemented a new parallel criterion, based on high plasma cell infiltration (≥5 plasma cells per field/5 HPFs, which we defined as CRIOAc Lyon's criterion). Patients were defined as infected, according to histopathological analysis, if at least one of the two criteria were present (≥5 plasma cells and/or PMNs per field/5 HPFs). For each slide, the three pathologists had to agree on the count assigned for PMNs, plasma cells and the presence or absence of fibrin, giant cells, granulation tissue and necrosis.

Descriptive statistics were used to estimate the frequencies of the study variables, described as percentages (%) for dichotomous variables and as the median (interquartile range (IQR)) for continuous values.

## Results

The study included 25 patients diagnosed with *C. acnes* chronic BJI from 2009 to 2013. The tissues had been collected during surgery in all cases. The corresponding samples (n=25) included 17 (17/25, 68%) samples addressed as bone and synovial samples, 7 (7/25, 28%) samples addressed as bone samples only and 1 (1/25, 4%) addressed as synovial samples. Of the 25 patients, 3 (3/25, 12%) had radiographic signs of prosthetic loosening only, 5 (5/25, 20%) had chronic pain only, 5 (5/25, 20%) had chronic BJI without associated clinical signs of infection, and 12 (12/25, 48%) patients had signs of infection at presentation (among them, all patients had chronic pain, 7 patients had local redness, 4 patients had purulent collection, 2 patients had fever (≥ 38.3°C) and 3 patients had sinus tract). The median delay between previous surgery and current diagnosis was 322 (179-786) days. The type of implant was more often prosthetic joint (16/25, 64%), and the type of surgery was more often debridement with implant removal (22/25, 88%). Clinical data are presented in Table 1.

**Figure 1:**
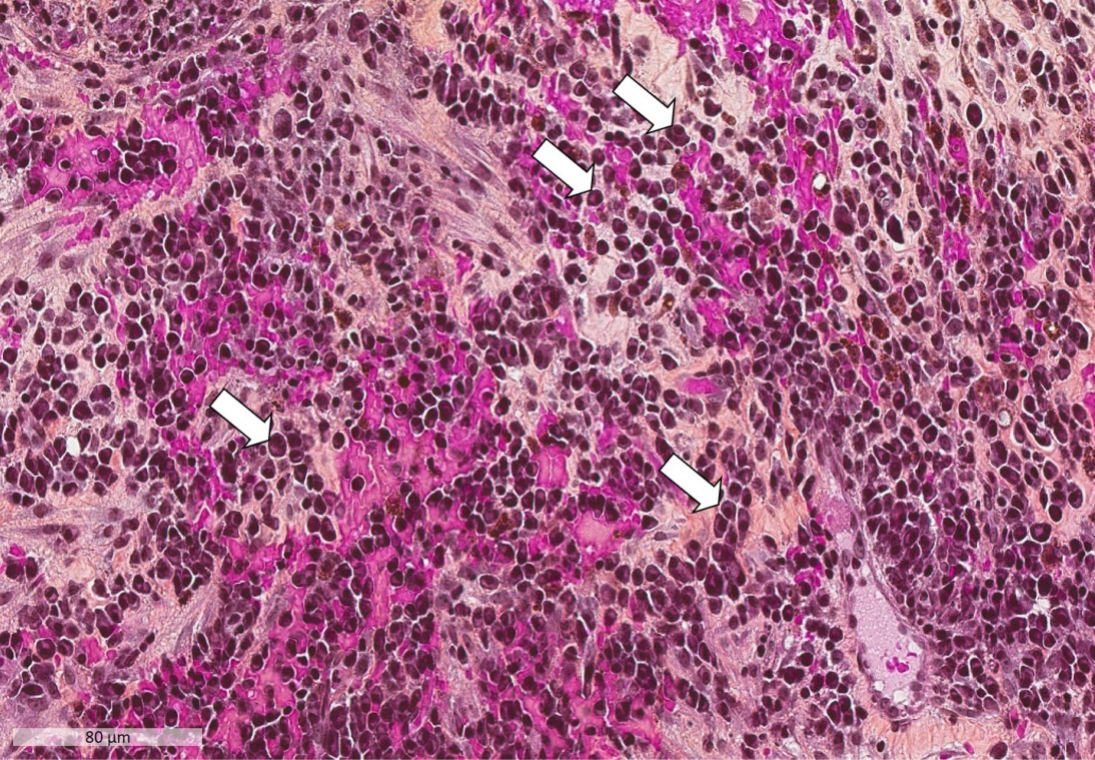
Plasma cell infiltration into synovial tissue (Hematoxylin-Eosin-Safran; x200 magnification). Numerous plasma cells are present. The white arrows indicate some of the plasma cells: they consist of an eccentric nucleus with heterochromatin in a characteristic cartwheel or clock face arrangement, surrounded by an abundant basophilic cytoplasm.

Of the 25 patients included in the study, 4 patients were not included in the final histopathological analysis (3 patients with only one *C. acnes* positive culture and 1 patient for which specimens were not analysable due to the sampling conditions and poor buffered formalin fixation; see Table 2). Histopathological inflammation criteria were collected as described above. According to Mirra's histopathological criterion, positive histopathology was observed in 12 (12/21, 57.1%) cases only. In 11 (11/12, 91.7%) cases PMNs were associated with plasma cells (≥5 plasma cells per field/5 HPFs in 10 cases, 2-5 plasma cells per field/5 HPFs in 1 case). Considering the 9 (9/21, 42.9%) cases with negative histopathology according to Mirra's criterion, high plasma cells infiltration (≥5 plasma cells per field/5 HPFs) was observed in 5 (5/9, 55.6%) cases (Figure 1), low plasma cells infiltration (2-5 plasma cells per field/5 HPFs) in 4 (4/9, 44.4%) cases, and fibrin, granulation tissue, giant cells and necrosis in 6 (6/9, 66.7%), 4 (4/9, 44.4%), 3 (3/9, 33.3%) and 1 (1/9, 11.1%) cases respectively. When there was ≥5 plasma cells per field/5 HPFs, the plasma cell infiltration patterns were not different depending on the tissue. In both sample types, there was diffuse and abundant plasma cell infiltration, as seen in Figure 1. However, in the bone samples, the plasma cell infiltration was present in contact with the cortex but also in the bone marrow. For the 7 samples addressed as bone samples only, Mirra's criterion was positive in 2 (2/7, 28.6%) cases, while CRIOAc Lyon's criterion was positive in 6 (6/7, 85.7%) cases. For the 9 patients with osteosynthesis implant, Mirra's criterion was positive in 4 (4/9, 44.4%) case, while CRIOAc Lyon's criterion was positive in 8 (8/9, 88.9%) cases. For the 12 patients with prosthetic implants and analysable samples, Mirra's criterion was positives in 8 (8/12, 66.7%) cases and CRIOAc Lyon's criterion in 7 (7/12, 58.3%) cases. Finally, of the 21 cases analysed, 15 (15/21, 71.4%) had abundant plasma cell infiltration. Adding abundant plasma cell infiltration (i.e. ≥5 plasma cells per field/5 HPFs which we defined as CRIOAc Lyon's criterion) to Mirra's criterion has restored histopathological diagnosis of PJIs and FRIs for a total of 5 (5/21, 23.8%) patients out of the 21. Of note, concerning the 3 patients with a single positive culture to *C. acnes*, none of them fulfilled Mirra's criteria, two had low plasma cell infiltration (one with <2 plasma cells per field/5 HPFs, and one with 2-5 plasma cells per field/5 HPFs) and one had high plasma cell infiltration (≥5 plasma cells per field/5 HPFs). These data are presented in Table 2.

## Discussion

*C. acnes* is well recognized as an orthopedic pathogen, sometimes difficult to detect 1, which can cause devastating complications after a prosthetic replacement surgery and have many clinical presentations 17. Acute BJIs, frequently associated with PMNs recruitment and purulence, are usually caused by staphylococci, streptococci and Gram-negative bacteria 1, 6, 14. *C. acnes*, considered as a slow-growing bacterium, is not frequently involved in acute BJIs, but more commonly implicated in chronic late infections, especially chronic implant-associated infections and sometimes only “colonization” potentially associated with prosthesis loosening 17, 18. The diagnosis of *C. acnes* BJI can be missed 11, and is frequently delayed, leading to an important cause of implant loosening, chronic pain and treatment failure 12, 13. The particularity of this germ is that it can lead to infections without inflammatory signs (prosthesis loosening without local inflammatory signs, sinus tract, or systemic symptoms of infection) and without elevation of C-reactive protein or the erythrosedimentation rate 1. In chronic PJIs, the Mirra's histopathological criterion used in the ICM 2018 3, 4, corresponding to ≥5 PMNs per field/5 HPFs, could fail to diagnose chronic PJIs 6, 9, 10. In our study, only 12 (12/21, 57.1%) of the 21 patients with chronic BJI due to *C. acnes* fulfilled Mirra's histopathological criterion. Adding the CRIOAc Lyon's criterion (i.e. ≥5 plasma cells per field/5 HPFs, 400× objective) permitted to restore histopathological diagnosis of infection in 5 (5/21, 23.8%) cases of patients without PMN infiltration.

Studies about histopathological diagnosis of BJIs mostly focused on the presence of PMNs. The ICM 2018 suggested that the commonly accepted threshold of 5 PMNs per field is too high for the diagnosis of all cases of arthroplasty infections (hip and knee) 3, 4, 9, as Pace et al. found less than 5 PMNs in 40% of cases when coagulase-negative staphylococci and *C. acnes* were isolated 19. Similarly, Bori et al. also reported that *C. acnes* and coagulase-negative staphylococci can cause a PJI with a PMN infiltration rate below five 6. Moreover, Kashima et al. noted that all cases of aseptic loosening contained fewer than 2 PMNs per HPFs 9. In our study, concerning the 3 cases in which “aseptic loosening” was finally found to be a *C. acnes* PJI, histopathology had low sensitivity to predict the presence of microorganisms. This fact was highlighted by some authors 10 and could be explained by the lack of adequate histopathological diagnostic criteria for PJIs due to *C. acnes*. These criteria may not be the same as those used for common bacterial PJIs (such as *Staphylococcus aureus*, or *Streptococcus* spp.). Indeed, many patients with suspected aseptic loosening have misdiagnosed PJI, as demonstrated by some authors 20.

However, none of these studies described the presence or absence of plasma cells in periprosthetic tissue, while this could be an element of the diagnosis. Indeed, plasma cells are the final step in maturation of B cell line, and part of adaptive immunity 21. In pathology, they are mainly present in chronic inflammatory reactions, often accompanied by lymphocytes 14, 22. In contrast, PMNs are part of the innate immunity, and are mainly recruited in acute inflammation 23. However, it is possible to find both plasma cells and PMNs in the same inflammatory reaction, which is explained by the fact that acute inflammation can evolve into prolonged subacute and chronic inflammation when the initial pathogen persists in tissues 14, 24. Due to the physiopathology of plasma cell inflammatory reaction, it is not surprising that the immune reaction caused by *C. acnes* could be mediated by plasma cells. Only one case reports the presence of plasma cell inflammation in a chronic indolent fracture-related tibial infection due to *C. acnes* 25.

We proposed a threshold of at least 5 plasma cells per field in at least 5 HPFs, which appeared to be a better diagnostic criterion (15/21 cases, 71.4%) than Mirra's criterion (12/21 cases, 57.1%) for defining chronic BJIs due to *C. acnes* in our study. Indeed, this threshold corrected the histopathological diagnosis for 5 (5/9, 55.5%) cases that had not been diagnosed as chronic BJI using Mirra's criterion. In addition, in this series, CRIOAc Lyon's criterion corrected more histopathological diagnosis than Mirra's criterion: i) when the samples were addressed as bone only (85.7% and 28.6% respectively); and ii) for patients with osteosynthesis, wherein the difference also seems to be particularly important (88.9% and 44.4% respectively).

However, plasma cells are not specific of BJIs caused by *C. acnes*, and differential diagnoses exist as inflammatory arthritides or plasma cell neoplasms 22, 24. Indeed, histopathological analysis and the presence of plasma cells in samples of patients with suspected chronic BJI can consequently evoke the presence of *C. acnes*, but must be part of a multidisciplinary approach, and should be correlated with other biological and clinical criteria.

In the 12 cases with at least 5 PMNs per field in 5 HPFs, fibrin was present in 10 (10/12, 83.3%) cases and granulation tissue in 9 (9/12, 75%) cases. In the event of a possible diagnostic score including plasma cells, these markers could perhaps play a secondary role. In contrast, the presence of giant cells and necrosis does not seem to be related to PMNs infiltration. However, despite adding CRIOAc Lyon's criterion to Mirra's criterion, 4 (4/21, 19%) cases were still considered histopathologically negative. Interestingly, for these 4 cases, all of them had an infiltration of 2-5 plasma cells/5 HPFs, 3 (3/4, 75%) had fibrin and 2 (2/4, 50%) had granulation tissue. In addition, among the 3 patients with chronic PJI, who were not included in the final histopathological analysis due to the presence of a single *C. acnes* positive culture, Mirra's criterion was negative in all cases. In contrast, the CRIOAc Lyon's criterion was positive in 1 (1/3, 33.3%) case, low 2-5 plasma cells/5 HPF was present in 1 (1/3, 33.3%) case, granulation tissue in 3 (3/3, 100%) cases, and fibrin in 2 (2/3, 66.6%) cases. It reinforces the hypothesis that a single histopathological criterion, for the diagnostic of chronic BJI, may not be enough, and perhaps, a combination of several criteria could better correct histopathological diagnosis.

Recently, Heim et al. have demonstrated that granulocytic myeloid-derived suppressor cells (G-MDSCs) represent the predominant leukocyte population in patients with PJIs. This accumulation of G-MDSC could support the chronicization of infection by preventing the proinflammatory and antimicrobial action of effector immune cells 26. In our study, the plasma cell infiltration, present in 15 (15/21, 71.4%) cases of infection due to *C. acnes*, could correspond to the late stage of inflammation in BJIs. A better understanding of the microenvironment of BJIs is therefore necessary to enable new immunomodulated therapeutic approaches.

To our knowledge, this is the first series of cases describing that plasma cells are present in BJIs due to *C. acnes*. Until now, no plasma cell threshold has been proposed for histopathological diagnosis of chronic BJIs due to *C. acnes*. In addition, in these infections the role of pathologists can be important, because the histopathological analysis is easy and requires a unique inexpensive standard staining by Hematoxylin-Eosin (HE), without immunohistochemistry examination.

To conclude, in our study and in literature, Mirra's criterion is not an adequate criterion to defining chronic BJIs 6, 9, 10, 19. However, adding CRIOAc Lyon's criterion based on plasma cells infiltration, restored some histopathological diagnosis of BJIs due to *C. acnes* when chronic BJI was clinically suspected. The presence of plasma cell infiltration should alert clinicians and pathologists of a possible chronic BJIs, since the criteria currently used are not appropriate. Further studies are needed to confirm the threshold of 5 plasma cells in each field/5 HPFs, to determine if PJIs and FRIs caused by others slow-growing pathogens could be associated with plasma cell infiltration, and to clarify if adding CRIOAc Lyon's criterion could be useful in the diagnosis of septic versus aseptic implant loosening.

## Figures and Tables

**Table 1:** Clinical data of patients with *C. acnes* BJI

	Total population, n=25
**Gender, n (%)**	
Female	11 (44%)
Male	14 (56%)
**Median age, years (IQR)**	61.5 (40.5-75)
**Median delay between previous surgery and current diagnosis, days (IQR)**	322 (179-786)
**Type of implant**	
Prosthetic joint, n (%)	16 (64%)
Osteosynthesis, n (%)	9 (36%)
**Type of surgery**	
Debridement and implant removal, n (%)	22 (88%)
Debridement and implant retention, n (%)	3 (12%)

**Table 2:** Histopathological and microbiological data from patients with chronic BJI due to *C. acnes*

Patients	Type of implant/location	Samples	No. of positive intraoperative culture to C. acnes*	Sinus tract	PMNs /5 HPFs^**^	Infection according Mirra's criterion (≥ 5 PMNs/5 HPFs)	Plasma cells /5 HPFs^**^	Infection according CRIOAc Lyon's criterion (≥ 5 Plasma cells /5 HPFs)	Diagnosis restored by adding CRIOAc Lyon's criterion to Mirra's criterion	Granulation tissue	Fibrin	Necrosis	Giant cells
1	PJ/knee	bone/synovial	3	-	0	-	2-5	-	-	+	+	-	+
2	OS/shoulder	bone/synovial	8	-	≥5	I	≥5	I	-	-	+	-	+
3	PJ/hip	bone/synovial	3	-	EX	EX	EX	EX	EX	EX	EX	EX	EX
4	PJ/hip	bone/synovial	4	-	≥5	I	≥5	I	-	+	+	-	-
5	OS/parietal bone	bone	2	-	0	-	≥5	I	**+**	-	-	-	-
6	OS/parietal bone	bone	2	-	0	-	2-5	-	-	-	-	-	+
7	PJ/knee	bone/synovial	1***	-	0	-	2-5	-	-	+	+	-	-
8	PJ/hip	bone/synovial	1***	-	<2	-	<2	-	-	+	+	-	-
9	PJ/knee	bone/synovial	2	-	<2	-	2-5	-	-	-	+	-	+
10	PJ/hip	bone/synovial	7	-	≥5	I	≥5	I	-	+	+	-	-
11	PJ/hip	bone/synovial	5	-	≥5	I	<2	-	-	+	-	-	-
12	PJ/hip	bone/synovial	5	+	≥5	I	2-5	-	-	+	+	-	-
13	PJ/knee	bone/synovial	4	-	≥5	I	≥5	I	-	+	+	-	+
14	PJ/hip	bone/synovial	6	-	≥5	I	≥5	I	-	-	+	-	+
15	PJ/hip	bone/synovial	5	-	<2	-	2-5	-	-	+	+	-	-
16	PJ/knee	bone/synovial	2	-	<2	-	≥5	I	**+**	-	+	-	-
17	PJ/hip	bone/synovial	3	-	≥5	I	≥5	I	-	+	+	-	+
18	PJ/hip	synovial	1***	+	0	-	≥5	I	**+**	+	-	-	-
19	PJ/hip	bone/synovial	3	-	≥5	I	≥5	I	-	+	+	-	-
20	OS/femur	bone/synovial	6	-	≥5	I	≥5	I	-	+	+	+	-
21	OS/ankle	bone	7	-	0	-	≥5	I	**+**	-	-	-	-
22	OS/femur	bone	5	-	2-5	-	≥5	I	**+**	+	+	+	-
23	OS/femur	bone	2	-	0	-	≥5	I	**+**	+	+	-	-
24	OS/humerus	bone	4	-	≥5	I	≥5	I	-	+	-	-	-
25	OS/femur	bone	4	+	≥5	I	≥5	I	-	-	+	-	-

HPFs: high-power fields; I: Infected; -: absence; +: presence; EX: excluded; PJ: Prosthetic joint; OS: Osteosynthesis. ^*^All positive cultures for *C. acnes* were monomicrobial and performed on separate deep samples collected intraoperatively from the site of infection. ^**^PMNs per field/5 HPFs and plasma cells per field/5 HPFs with 4 possible scores: 0, <2, 2-5, ≥5. Patients were considered as infected, if they had ≥5 plasma cells and/or PMNs per field/5 high-power fields. *** Patients with only one *C. acnes* positive culture were not included in the final histopathological analysis, in order to analyse the proven* C. acnes* infections only.

## Abbreviations

BJI: Bone and joint infection
PJI: prosthetic joint infection
FRI: fracture-related chronic infection
ICM: International Consensus Meeting
*C. acnes*: *Cutibacterium acnes*
FFPE: formalin-fixed paraffin embedded
PMNs: Polymorphonuclears
HPF: high power fields
CRIOAc Lyon: Centre de Référence des Infections Ostéo-Articulaires Complexes de Lyon
G-MDSCs: granulocytic myeloid-derived suppressor cells
